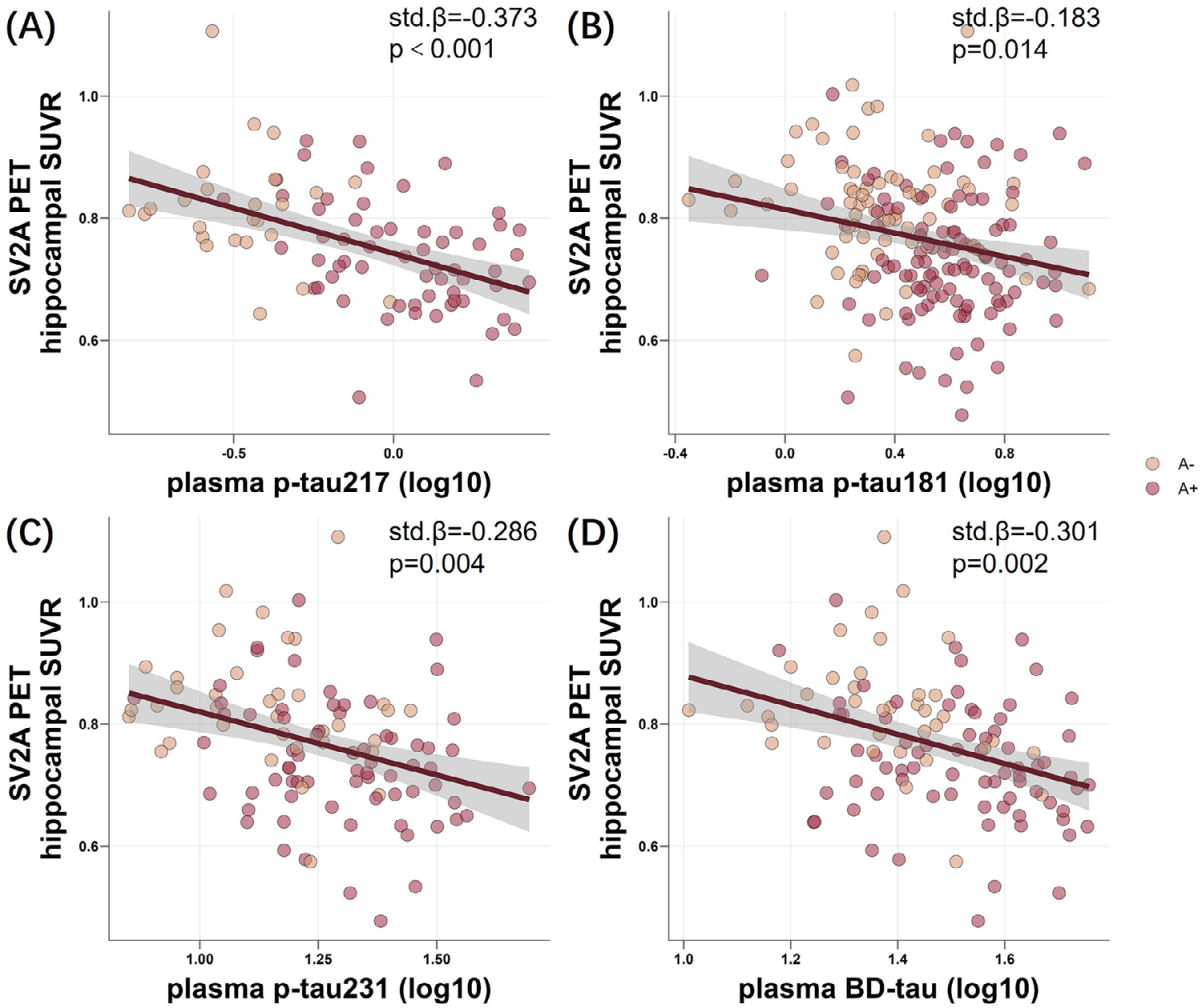# The association between tau‐related fluid biomarkers and synapse‐associated biomarkers in Alzheimer's disease

**DOI:** 10.1002/alz70856_101974

**Published:** 2025-12-24

**Authors:** Mengjie Wang

**Affiliations:** ^1^ Huashan Hospital, Fudan University, shanghai, shanghai, China

## Abstract

**Background:**

Synaptic damage is a major cause of cognitive decline in Alzheimer's disease (AD) patients. SV2A, which is synaptic vesicle protein 2A, have been confirmed as synaptic injury biomarkers for AD. We aim to investigate the mechanisms underlying the association between synaptic damage and tau pathology.

**Methods:**

We included 239 subjects (age: 68.7 ± 7.3; female: 66.1%) from the Huashan cohort in Shanghai, China. Subjects from the Huashan cohort underwent at least one SV2A PET scan (18F‐SynVesT‐1). We first used the t‐test method to compare the differences in synaptic biomarkers and various plasma tau biomarkers between the amyloid PET negative (A‐) and positive (A+) groups. We then used a GLM model to analyze the baseline synaptic biomarkers in relation to plasma tau biomarkers. Both the GLM and mediation models were adjusted for age, sex, and years of education.

**Results:**

Synaptic injury were significantly increased in the amyloid PET‐positive group. Among the tau‐related plasma biomarkers, *p*‐tau217 showed a significant association with the synaptic biomarkers. The effects of plasma *p*‐tau217 on the cross‐sectional SV2A PET hippocampal SUVR values were mediated by FBP Centiloid and MK6240 MetaTEM‐ROI SUVR values, while the effects on cerebrospinal fluid (CSF) GAP43 were mediated by CSF Aβ42 and CSF *p*‐tau181.

**Conclusion:**

This study confirms that phosphorylated tau pathology is a critical pathway for synaptic damage. Plasma *p*‐tau217 is closely associated with peripheral and brain synaptic changes, showing potential as a plasma biomarker for early screening and prediction of AD synaptic injury pathology.